# Substrate diffusion electrodes allow for the electrochemical hydrogenation of concentrated alkynol substrate feeds

**DOI:** 10.1016/j.isci.2025.111789

**Published:** 2025-01-10

**Authors:** Jonas Wolf, Fatima Shahrour, Zafer Acar, Kevinjeorjios Pellumbi, Julian Tobias Kleinhaus, Leon Wickert, Ulf-Peter Apfel, Daniel Siegmund

**Affiliations:** 1 Department of Electrocatalysis, Fraunhofer Institute for Environmental, Safety and Energy Technology UMSICHT, 46047 Oberhausen, Germany; 2 Technical Electrochemistry, Faculty of Chemistry and Biochemistry, Ruhr University Bochum, 44801 Bochum, Germany

**Keywords:** Chemical engineering, Electrochemistry, Engineering

## Abstract

Electrosynthesis has the potential to revolutionize industrial organic synthesis sustainably and efficiently. However, high cell voltages and low stability often arise due to solubility issues with organic solvents, while protic electrolytes restrict substrate options. We present a three-layered electrode design that enables the use of concentrated to neat substrate feeds. This design separates the organic substrate from the aqueous electrolyte using layers with varying porosity and hydrophilicity, ensuring precise reactant transport to the catalyst layer while minimizing substrate and electrolyte crossover. We demonstrate its effectiveness by semi-hydrogenating three alkynols with different hydrophobicities. For the semi-hydrogenation of 3-methyl-1-pentyn-3-ol in pure form, we achieved 65% faradaic efficiency at 80 mA cm^−2^. Additionally, semi-hydrogenation of neat 2-methyl-3-butyn-2-ol on palladium showed a faradaic efficiency for semi-hydrogenation of 36%, that was stable for 22 h. This design could be pioneering the electrochemical valorization of neat substrates, reducing the need for extensive downstream processing.

## Highlights

• 3-layered electrode design enables electrochemical conversion of neat substrates
• The substrate diffusion electrode reduces substrate and electrolyte crossover
• High faradaic efficiencies (up to 79%) achieved in semi-hydrogenation reactions

## Graphical abstract

**Figure:**
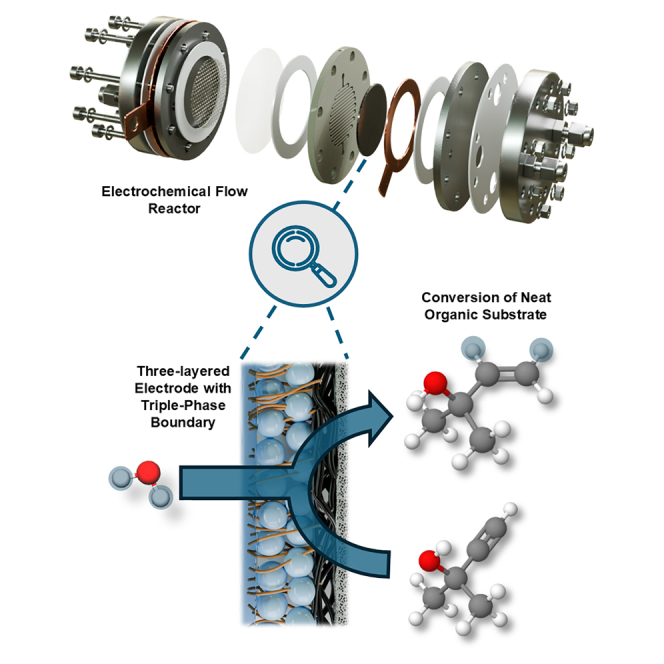

## Introduction

The use of renewable electricity to drive electrochemical processes and the flexibility to control reactions through the electrochemical potential make electrosynthesis a valuable tool for the future of the chemical industry.^1^^,^^2^^,^^3^^,^^4^^,^^5^^,^^6^ However, real-world electrochemical processes face significant challenges and costs, particularly due to the use and necessary separation of solvents and conductive electrolyte salts, which complicate product stream workup.^7^^,^^8^^,^^9^ A key approach for the highly efficient conversion of organic substrates with low cell resistances is solid polymer electrolyte (SPE) electrolysis, in which one central layer, mostly an ionomer membrane, functions as electrolyte and half-cell separator simultaneously.^10^^,^^11^^,^^12^ Systems using pure substrate feeds, e.g., SPE systems, face a number of problems: the crossover between electrolyzer compartments necessitates downstream purification of the product stream, especially in the case of water-soluble substrates when extrapolated to industrial applications.^13^^,^^14^^,^^15^ Moreover, under the demanding conditions of neat organic substrates, cell components—especially polymer electrolyte membranes—are prone to decomposition, which introduces impurities and severely impacts long-term stability.^15^^,^^16^^,^^17^^,^^18^^,^^19^^,^^20^ Additionally, the poor solubility of common organic substrates in aqueous electrolytes poses a challenge, as using organic solvents increases cell resistances.^15^

To address these solubility limitations, a few current approaches, such as the palladium membrane reactor (PMR), separate the organic substrate in one compartment while allowing reactive species from an adjacent compartment to participate in the reaction.^6^^,^^15^^,^^21^ However, the mass transport of reactive species from the adjacent compartment to the catalyst layer can limit reaction rates, particularly at high substrate concentrations. In such cases, few or none of these reactive species are present in the substrate compartment itself. This severely restricts the range of substrates and concentrations that existing electrode concepts can handle when facilitating reactions of organic substrates with aqueous electrolytes.^6^^,^^12^^,^^14^^,^^21^^,^^22^ As electrochemical processes advance toward industrial-scale applications, overcoming these limitations is crucial to compete effectively with established thermocatalytic methods.

Herein, we propose a concept to valorize organic substrate feeds: the substrate diffusion electrode (SDE). It establishes a triple phase boundary (TPB) between a concentrated organic substrate feed, an electrolyte, and a catalyst, aimed at addressing the challenges in organic electrochemistry.^23^^,^^24^^,^^25^^,^^26^^,^^27^ The concept is inspired by our group’s extensive experience in optimizing gas diffusion electrodes (GDE),^23^^,^^24^^,^^25^^,^^26^^,^^27^ which are widely used in gas-phase reactions such as carbon dioxide valorization and fuel cells.^28^^,^^29^^,^^30^^,^^31^^,^^32^ Similar to the SDE, they create a TPB between the catalyst, electrolyte, and gas stream, precisely where the desired reaction occurs while preventing reservoir mixing.

Similar to GDEs, features like microporous layers and hydrophobic material treatments in the SDE are expected to help facilitate substrate transport to the catalyst layer and prevent flooding.^33^^,^^34^^,^^35^ Each layer and component of the SDE was thoroughly investigated to understand the interplay between TPB properties and the conversion of different organic substrates. Ultimately, the goal was to develop and demonstrate a versatile electrode concept that is flexible in both substrate and catalyst, thereby enabling the electrochemical valorization of neat organic substrates.

## Results and discussion

### Electrode architecture

**Figure 1:**
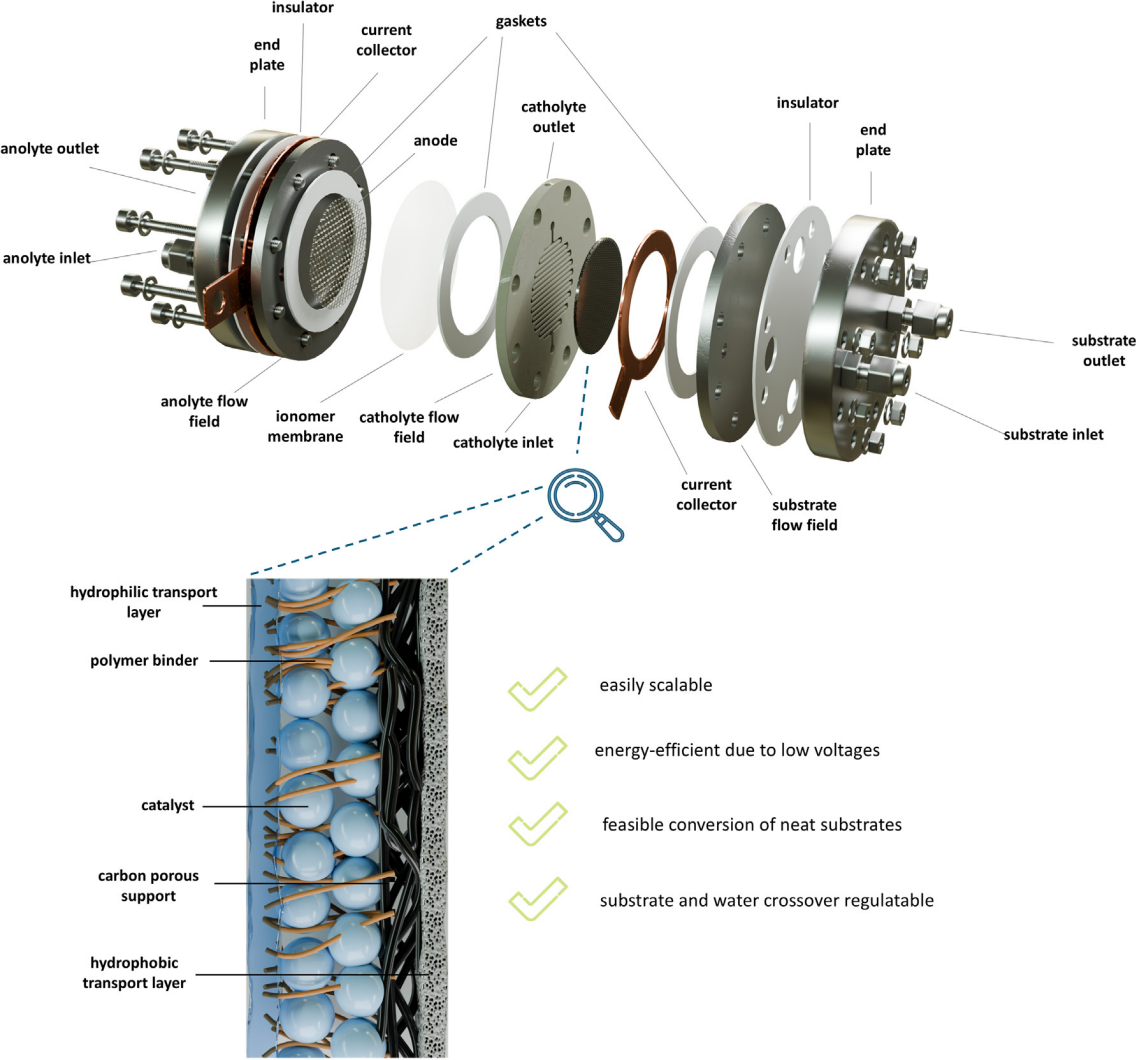
Structure of a substrate diffusion electrode (SDE) in an electrochemical flow reactor The SDE, that enables the conversion of concentrated and neat substrate feeds, consists of a middle layer with the catalyst, surrounded by hydrophilic and hydrophobic transport layers, that ensure controlled substrate and electrolyte transport to the catalyst layer, respectively, and mitigate crossover. The SDE is integrated into an electrochemical flow reactor with distinct anolyte, catholyte, and substrate compartments (see also Figure S1).

To explore the SDE, we employed electrochemical hydrogenation (EChH) as a model reaction due to our extensive research on such conversions.^15^^,^^19^^,^^25^^,^^26^^,^^27^^,^^36^^,^^37^ The tasks of the SDE are a controlled transport of both substrate and electrolyte to the catalyst layer. In the case of EChH, the relevant species that must be transported to the catalyst layer are the organic substrate and hydrogen equivalents forming the active species, which are free protons or adsorbed hydrogen atoms depending on the reaction pathway.^38^^,^^39^^,^^40^^,^^41^^,^^42^ For our studies, a thin ionomer membrane was used as the hydrophilic transport layer to facilitate electrolyte movement to the catalyst layer (Figure 1). The catalyst layer, in direct contact with the ionomer membrane, is coated onto a porous carbon support to maximize the active surface area.

Another critical component of the SDE is the hydrophobic separation layer on the substrate-facing side. This layer prevents the crossover of water and electrolyte ions from the adjacent catholyte compartment. By adjusting the membrane thickness and pore size, we balanced the necessary substrate transport to the catalyst layer with the need to minimize electrolyte crossover. Initial material screenings identified suitable separation layers made from hydrophobic polytetrafluoroethylene (PTFE) (Table S1). This study demonstrates how the SDE can effectively enable electrochemical hydrogenation (EChH) of pure organic substances across various combinations of substrates and catalysts.

### Employed system components

**Figure 2:**
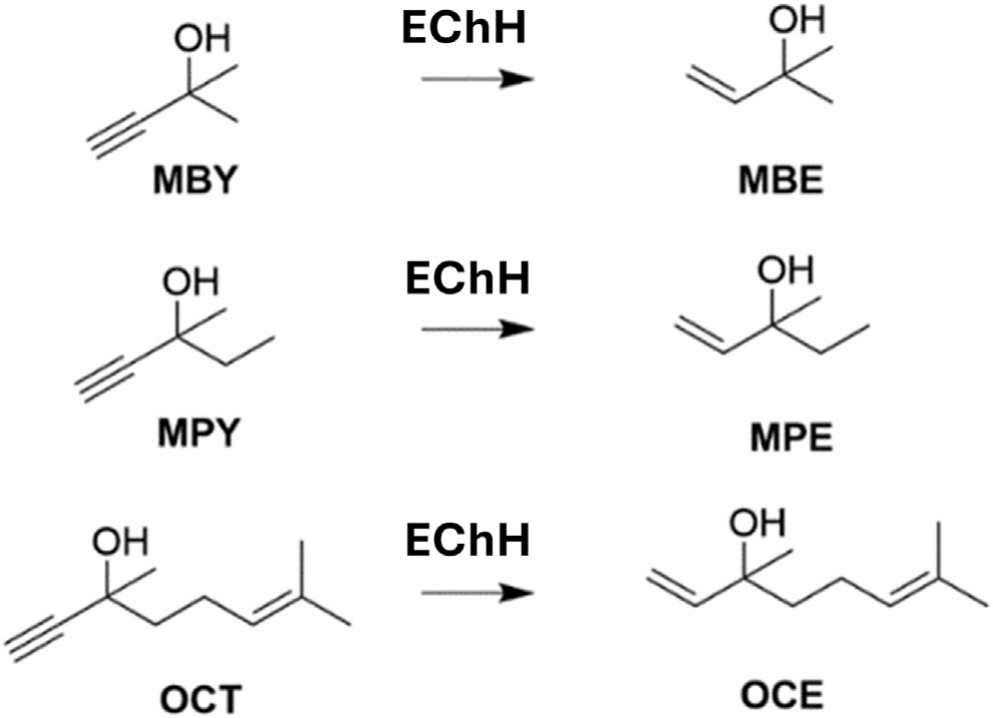
Organic substrates for the electrochemical conversion with the substrate diffusion electrode and their semi-hydrogenation products MBY: 2-methyl-3-butyn-2-ol; MBE: 2-methyl-3-buten-2-ol; MPY: 3-methyl-1-pentyn-3-ol; MPE: 3-methyl-1-penten-3-ol; OCT: 3,7-dimethyl-6-octen-1-yn-3-ol; OCE: 3,7-dimethylocta-1,6-dien-3-ol.

We tested three alkynol substrates with varying physicochemical properties to validate the electrode’s versatility: 2-methyl-3-butyn-2-ol (MBY), 3,7-dimethyl-6-octen-1-yn-3-ol (OCT), and 3-methyl-1-pentyn-3-ol (MPY) (Figures 2 and S8–S10). MBY is a precursor to the vitamin A and E synthon 2-methyl-3-buten-2-ol (MBE)^25^^,^^27^^,^^36^^,^^37^ and is fully miscible with water. MPY and OCT have water solubilities of 112 g L^−1^ and less than 1 g L^−1^, respectively, due to their different carbon chain lengths.^43^^,^^44^

To address the diverse substrate-catalyst combinations in electro-organic conversions, we aimed to develop an electrode concept that is independent of both catalyst and substrate. We tested this concept by hydrogenating three different substrates using three distinct EChH catalysts: palladium (a benchmark catalyst), silver,^27^ and the pentlandite Fe_3_Ni_6_S_8_ (demonstrated as an effective catalyst in previous studies).^25^^,^^26^^,^^36^^,^^37^^,^^45^ The catalysts were spray-coated onto carbon supports with polymer binders using established protocols^25^ and then hot-pressed onto a thin ionomer membrane.

The SDE includes a hydrophobic substrate-facing layer, a hydrophilic electrolyte-facing layer, and a carbon porous transport layer (Figure 1). In the configuration used as the starting point for further trials, Nafion N212 was employed as the hydrophilic layer, while pentlandite Fe₃Ni₆S₈ with 10 wt % PTFE on H23i2 carbon paper served as the middle layer. The oxygen evolution reaction (OER) in an alkaline medium was chosen as the well-established, efficient, and waste-free anode reaction, with an ionomer membrane used to separate the anolyte and catholyte compartments.^25^^,^^27^^,^^36^ All SDE components—the hydrophobic layer, the hydrophilic layer and the carbon porous transport with the catalyst layer—were subjected to a brief screening of materials to determine feasible configurations for basic function and to serve as starting points for further investigations.

**Figure 3:**
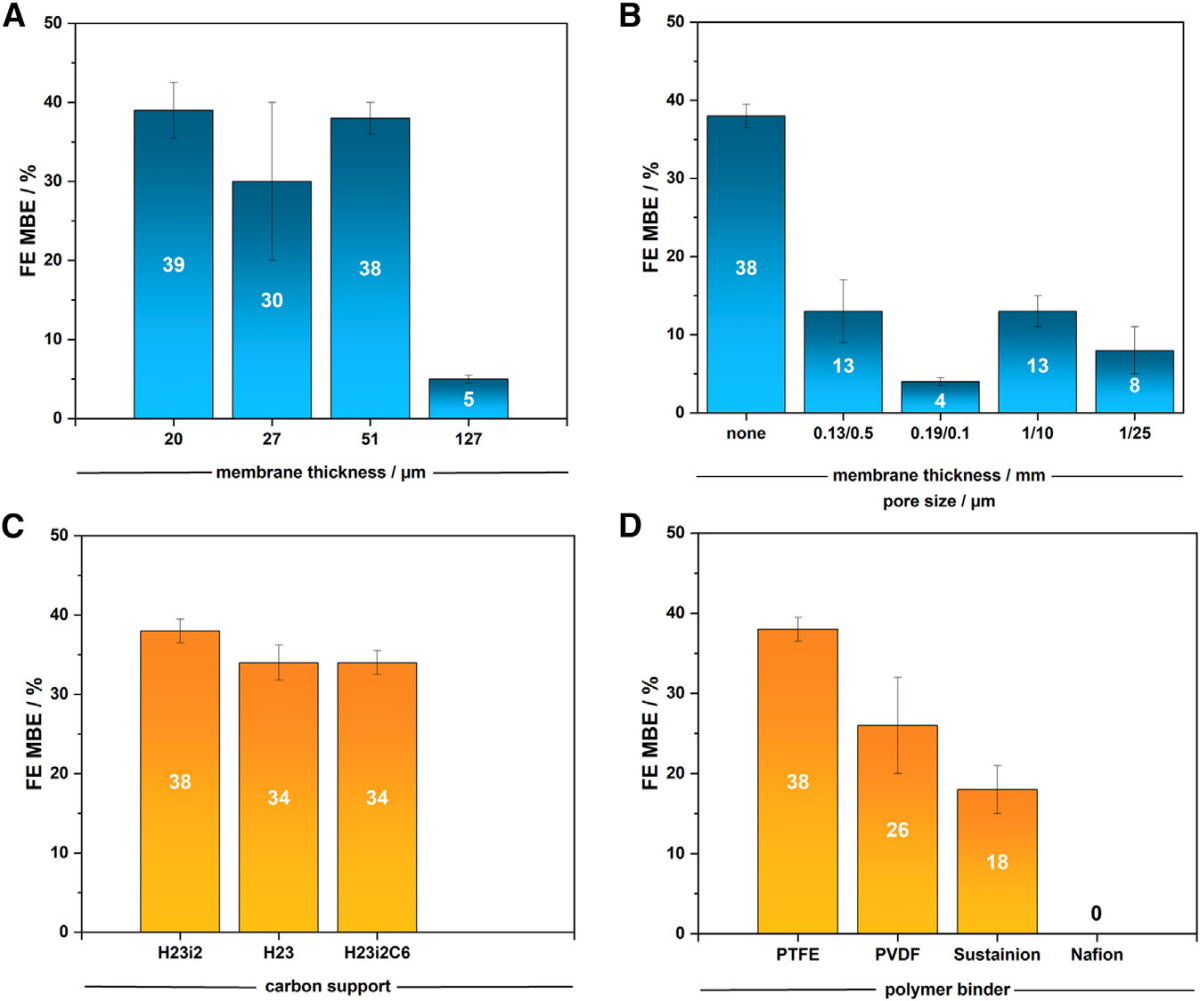
Assessment of electrochemical performance upon varying substrate diffusion electrode components (A–D) Faraday efficiency (FE) of the electrolysis of neat 2-methyl-3-butyn-2-ol (MBY) to 2-methyl-3-buten-2-ol (MBE) in an electrochemical flow reactor with a substrate diffusion electrode (SDE) varying ionomer membrane (A) as the hydrophilic transport layer steering the electrolyte transport to the catalyst layer and mitigating substrate crossover, PTFE-membrane (B) as the hydrophobic transport layer steering the substrate transport to the catalyst layer and mitigating water crossover, porous carbon support (C) and polymer binder (D) (Nafion-bound electrodes resulted in decomposition of the catalyst layer; room temperature; v_flow_ = 12 mL min^−1^, j = 80 mA cm^−2^; electrolysis duration: 1 h; data were measured as duplicates and are represented as mean ± σ; see also Tables S1–S3).

Our analysis of the hydrophobic layer (Figure 1) revealed that PTFE membranes thinner than 100 μm did not effectively reduce electrolyte crossover to the substrate compartment, regardless of pore size. Conversely, membranes thicker than 1 mm with pore sizes below 5 μm significantly restricted substrate transport, resulting in minimal product formation. We therefore targeted materials within these limits that minimized electrolyte crossover while maintaining high Faraday efficiency (FE) for semi-hydrogenation, compared to configurations without a PTFE layer (Figure 3).

Subsequent measurements were performed without a hydrophobic layer and with two different PTFE layers: **M**_**dense**_ (an expanded PTFE membrane with a filtration efficiency of 99% for 0.5 μm and 130 μm thickness) and **M**_**wide**_ (a membrane with 10 μm average pore size and 1 mm thickness) (Table S1). Contact angles on **M**_**dense**_ and **M**_**wide**_ were 137° and 116°, respectively, confirming a more feasible transport of water and substrate for **M**_**wide**_ compared to **M**_**dense**_. A screening of further materials combined with computational simulations of substrate and electrolyte transport through the hydrophobic transport layer will be conducted in further studies to minimize crossover while optimizing electrolytic performance.

The Nafion membrane within the SDE was employed to manage water and potassium ion crossover from the catholyte to the substrate compartment (Figure 3; Table S2). The crossover of cations through ionomer membranes is directly connected to the transport of water due to the ions' solvation shell, e.g., leaving hydroxide as a counter ion on the other side of the membrane.^46^ Along this line, water crossover is quantified as the percentage of water in the product mixture after 1 h of electrolysis, while potassium crossover is measured as the proportion of charge equilibrated by the transport of potassium ions through the SDE's thin ionomer membrane. Thinner membranes increased undesired water crossover, while also facilitating proton transport at lower voltages. Conversely, thicker membranes generally reduced water crossover but required higher voltages.^47^ The thickest membrane (127 μm) gave the lowest faradaic efficiency for MBY (FE_MBE_) of 5% with nearly 0% water crossover, while the thinnest membrane (20 μm) achieved the best FE_MBE_ of 39%, however, along the highest water crossover rate of 17%. Therefore, Nafion N212 with a thickness of 51 μm was selected for further use, since it gave relatively low water (7%) and potassium (19%) crossover.

Developing a robust catalyst layer capable of enduring the conditions associated with neat organic substrates while ensuring efficient semi-hydrogenation poses significant challenges due to the solubility and chemical stability of polymer binders.^15^^,^^16^^,^^17^^,^^18^ The stability and performance of the binder are influenced by its solubility and hydrophobicity. We evaluated four polymer binders differing in hydrophobicity and solubility—PTFE, PVDF, Nafion, and Sustainion XA-9—each at 10 wt % relative to the catalyst, supported on carbon paper for the EChH of MBY, MPY, and OCT (Figure 3; Table S3).^25^^,^^36^^,^^37^ Non-ionic and hydrophobic PTFE exhibited the highest faradaic efficiency (FE) for MBY, achieving 38%, followed by PVDF with 26%, and the ionomer Sustainion XA-9 with 18%. Nafion layers were unstable with neat MBY, leading to rapid delamination from the porous transport layer. Dynamic vapor sorption (DVS) experiments indicated that PTFE-based catalyst layers absorbed 50% more water than Nafion-based ones, potentially enhancing water and proton supply to the catalyst during operation. PTFE’s rapid water uptake, which prevented accurate contact angle measurements, supports these findings. For OCT and MPY, PTFE caused complete delamination of the catalyst layer within minutes, whereas Nafion-bound layers achieved FEs of 13% and 4%, respectively. These results highlight the need for precise tuning of SDE components to achieve desired conversion efficiencies. The choice of the porous transport layer impacts chemical stability, FE, and water crossover rates, which were thoroughly investigated (Table S3). Three carbon paper supports were compared: one with hydrophobic pretreatment (H23i2), one with hydrophobic pretreatment and an additional microporous layer (H23i2C6) below the catalyst layer, and a neat support (H23). All supports showed similar FEs of approximately 35%, indicating that support hydrophobicity and the microporous layer have a minor effect on electrochemical performance. However, the water crossover from the electrolyte to the substrate compartment decreased from 15% to 8% and 7% for H23, H23i2C6, and H23i2, respectively, due to their hydrophobic treatment. Nevertheless, the hydrophobic treatment led to rapid catalyst layer decomposition for substrates MPY and OCT. Consequently, H23i2 was used with PTFE for MBY and H23 with Nafion for MPY and OCT. The contact angle of the electrode surface increased from 120° to 130° upon coating with a Nafion-bound pentlandite catalyst layer in the case of H23 carbon paper as the porous support. For the hydrophobically treated H23i2, the contact angle increased from 132° to 143° when coating with the respective catalyst layer. This and the previously mentioned DVS results as well as the attempted contact angle measurement of the PTFE-bound layers reveal that PTFE macroscopically exerted a hydrophilizing effect on the electrode while Nafion exerted a macroscopically hydrophobizing effect. This suggests a better performance of Nafion-bound catalyst layers for non-polar and of PTFE-bound ones for polar substrates due to potentially facilitated interactions between substrate and catalyst layer, as it is confirmed by the results obtained in this study.

Scanning electrode microscopy (SEM) and energy-dispersive X-ray spectroscopy (EDX) (Figures S2–S6) revealed no relevant changes in the morphology or elemental composition of the exemplarily investigated Nafion-bound palladium catalyst layers upon electrolysis of neat MPY and OCT (Figures S2, S4, and S5). Merely, new oxygen and potassium bands, most likely originating from a film of potassium hydroxide from the adjacent electrolyte, were observed after electrolysis. For the PTFE-bound pentlandite catalyst layers, a stark decrease in the overall sulfur content was observed that can be traced back to the literature-known sulfur loss during electrolysis (Figures S3 and S6).^48^ Moreover, sulfur and fluorine, which is representative for the PTFE binder, appear more locally concentrated in the EDX-maps revealing a restructuring of the catalyst layer during electrolysis, likely going hand-in-hand with the changes in pentlandite composition.

### System configuration

**Figure 4:**
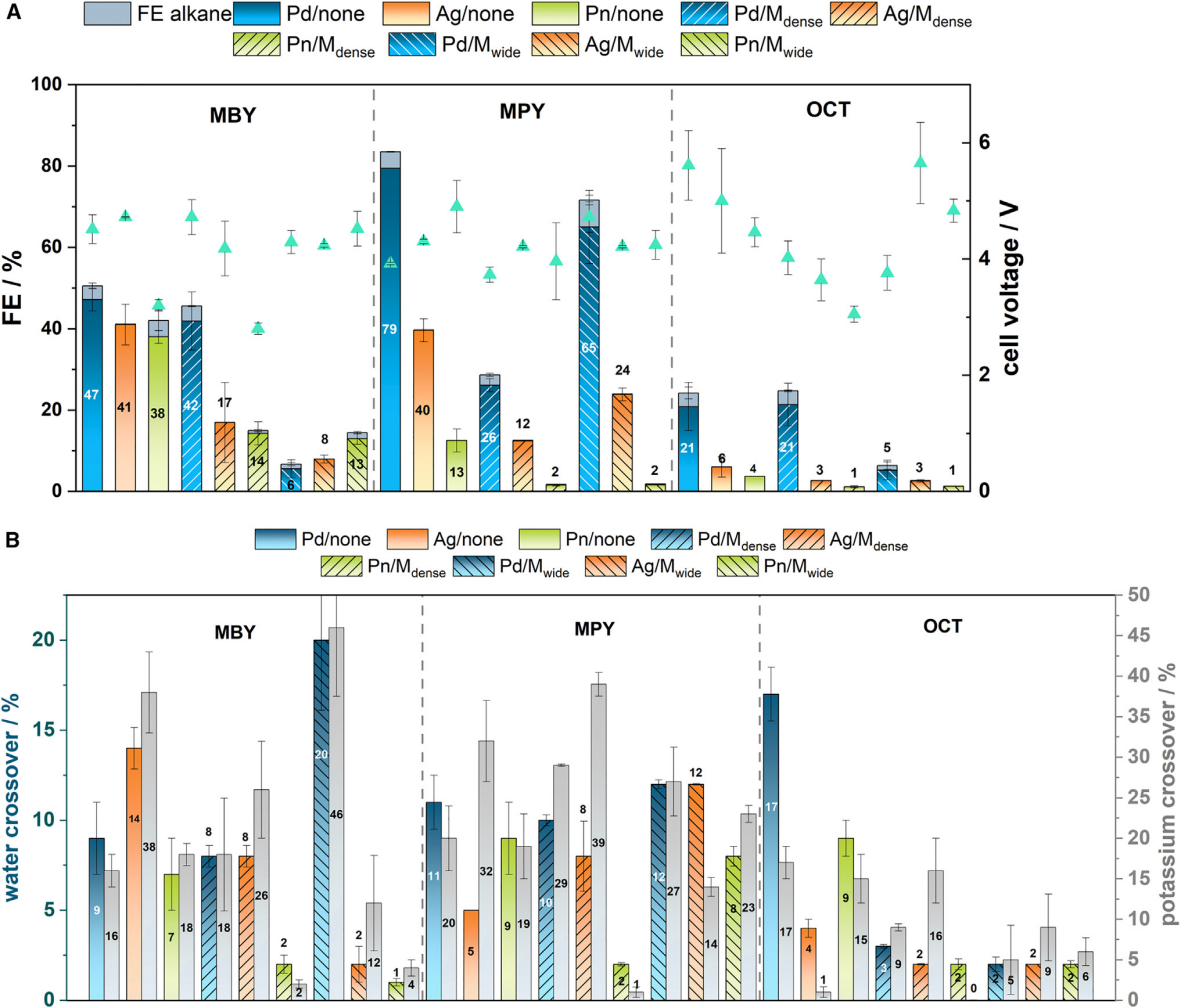
Influence of substrate diffusion electrode (SDE) properties, substrate and catalyst on the electrochemical semi-hydrogenation performance (A and B) Faraday efficiencies (FE) and cell voltages (A) of the formation of the respective alkene and alkane from 2-methyl-3-butyn-2-ol (MBY), 3-methyl-1-pentyn-3-ol (MPY) and 3,7-dimethyl-6-octen-1-yn-3-ol (OCT) as neat substrates as well as potassium crossover (given as the percentage of consumed charge equilibrated by the transport of a potassium ion) and water crossover data (given as the share of water in the product stream after 1 h electrolysis) (B) obtained from electrolysis in a flow reactor employing an SDE with varying catalysts and PTFE hydrophobic substrate transport layers (Pn = pentlandite Fe_3_Ni_6_S_8_; room temperature; v_flow_ = 12 mL min^−1^, j = 80 mA cm^−2^; electrolysis duration: 1 h; data were measured as duplicates and are represented as mean ± σ; see also Table S4).

After assessing the SDE components, various catalysts, substrates, and PTFE layers were tested to validate the SDE’s catalyst- and substrate-flexible performance in the hydrogenation of neat organics. The FEs for semi-hydrogenation, cell voltages, and crossover rates are detailed in Figure 4 and Table S4. Electrolysis was conducted for 1 h at a constant current density of 80 mA cm^−2^ of geometric surface area unless stated otherwise. The reactor was designed with three compartments to independently optimize the catholyte and anolyte conditions (Figure 1).

Palladium consistently demonstrated higher catalytic performance compared to silver and Fe₃Ni₆S₈, particularly for hydrophobic substrates and in the absence of a PTFE layer (Figure 3). For MBY, FEs were 47% for palladium, 41% for silver, and 38% for Fe₃Ni₆S₈, showing minimal variation. The given faradaic efficiency for the MBY EChH on palladium corresponds to a reaction rate of 4.9 mmol h^−1^ and a catalyst mass activity of 30.0 g_MBE_ g_cat_^−1^ h^−1^. In contrast, for MPY and OCT, palladium achieved elevated FEs up to 79% and 21%, respectively, corresponding to reaction rates of 8.3 and 2.2 mmol h^−1^ and to catalyst mass activities of 58.5 and 24.1 g_alkene_ g_cat_^−1^ h^−1^, respectively. For comparison, state-of-the-art protocols of the semi-hydrogenation of MBY in aqueous solution feature catalyst mass activities of 357.0 g_MBE_ g_cat_^−1^ h^−1^ at faradaic efficiencies of 58%.^49^ Silver reached only 40% and 6%, and Fe₃Ni₆S₈ reached 13% and 4%, for MPY and OCT, respectively. This trend is attributed to inherent differences in catalyst activity, as previously observed for the EChH of MBY.^25^^,^^27^^,^^36^^,^^37^ Electrolysis of MPY and OCT in ethanol-water mixtures in a conventional zero-gap electrolyzer using palladium-coated carbon paper electrodes only gave Faraday efficiencies of 50% and 9%, respectively (Table S5), accompanied by decomposition of the ionomer membrane, proving the superiority of the SDE. Palladium exhibited the highest FE for over-hydrogenation to the corresponding alkanes, while silver showed the lowest FE, aligning with previous findings. The tendency for over-hydrogenation to alkanes is highly dependent on the catalyst’s properties and remains unaffected by the use of PTFE layers in the SDE.

With the substrate-facing PTFE membrane **M**_**dense**_, which prioritizes minimization of water crossover, the FEs for MBY were 42% for palladium, 17% for silver, and 14% for Fe₃Ni₆S₈, showing only minor changes compared to the configuration without a PTFE membrane for palladium. This suggests that palladium’s flexibility in balancing hydrogen and substrate coverage on the catalyst aids in efficient EChH.^50^ Conversely, with **M**_**wide**_, which should primarily facilitate efficient substrate transport to the catalyst layer, an inverse trend was observed for MBY: FEs were 6%, 8%, and 13% for palladium, silver, and Fe₃Ni₆S₈, respectively. Overall, the presence of PTFE layers decreases efficiency for all substrates, with some exceptions. For MPY with palladium and **M**_**wide**_, the FE only slightly decreased from 79% to 65%. However, MBY and OCT exhibited significant decreases in efficiency with **M**_**wide**_ but minimal changes with **M**_**dense**_ for palladium-catalyzed EChH: MBY decreased from 47% to 42%, and OCT remained at 21%. The observed optimum combinations of substrate and PTFE membrane indicate that the hydrogen-to-substrate coverage ratio on the catalyst layer depends on the selected materials. Along the line of the previously given contact angles on the PTFE membranes, **M**_**dense**_ is expected to lower local substrate concentration and to increase local water concentration due to their inhibited transport to the adjacent compartments, while **M**_**wide**_ is expected to have the opposite effect. However, providing a conclusive relation between material selection, local reactant concentrations and system performance requires extended spectroscopic analysis, which is the object of extended ongoing studies.

Water and potassium crossover quantification showed no clear dependence on the catalyst and substrate, but the PTFE membrane significantly impacted these rates. Without a PTFE layer, the product stream contained about 7% water on average, which decreased to 2% with **M**_**dense**_ and 1% with **M**_**wide**_. Potassium crossover from the catholyte to the substrate compartment correlated with water crossover rates and varied significantly. For example, OCT had an average water crossover rate of 9%, while MBY and MPY had rates of 7% and 9%, respectively, with corresponding potassium crossover rates of 15%, 18%, and 20%. The observed trend may be due to the phase boundary between non-polar OCT and the polar electrolyte, which could hinder electrolyte crossover. No substrate crossover to the catholyte compartment was detected in any experiment *via* ^1^H-NMR spectroscopy.

The cell voltages observed in the experiments ranged from 2.80 to 5.65 V, with 3,7-dimethyl-6-octen-1-yn-3-ol (OCT) requiring higher voltages on average (4.47 V) compared to 2-methyl-3-butyn-2-ol (MBY) at 4.13 V and 3-methyl-1-pentyn-3-ol (MPY) at 4.33 V. The higher voltages for OCT can be attributed to additional overpotentials associated with its micelle transport and breakage, coupled with its very low water solubility, which impairs conductivity. MPY also exhibits a phase boundary with water in certain mixtures, which may contribute to the observed voltages. Despite these variations, the cell voltages remain competitive with established protocols for alkynol hydrogenation, demonstrating the advantages of using neat substrate conversions.^25^^,^^27^^,^^36^^,^^37^ Further system refinements, such as implementing a thinner middle compartment, could further reduce cell voltages and enhance the feasibility of the SDE. Future investigations will explore reactor configurations that integrate both cathodic and anodic SDEs to combine multiple value-added processes in a single setup.

### Operation at high current densities, differing substrate concentrations and long runtimes

**Figure 5:**
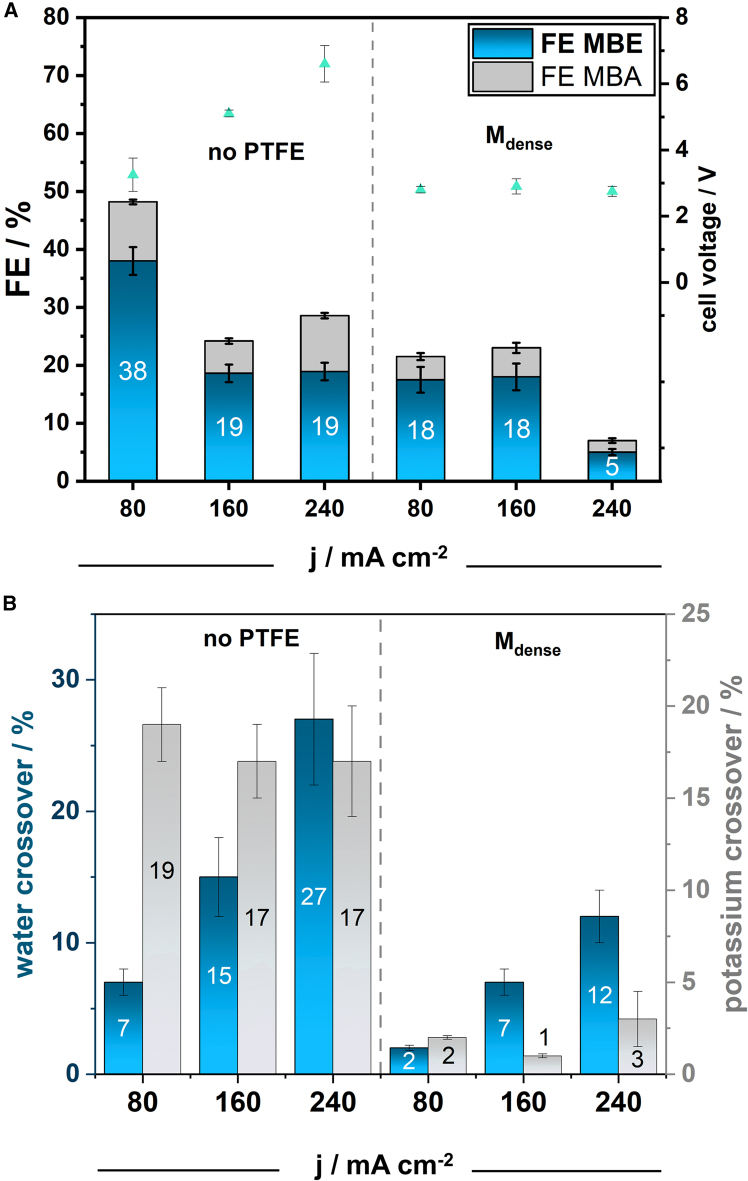
Enhancing current densities in the electrochemical hydrogenation with substrate diffusion electrodes (A and B) Faraday efficiencies (FE) for the formation of 2-methyl-3-buten-2-ol (MBE) and 2-methylbutan-2-ol (MBA), cell voltages (A), potassium crossover (given as the percentage of consumed charge equilibrated by the transport of a potassium ion) and water crossover data (given as the share of water in the product stream after 1 h electrolysis) (B) obtained from the electrolysis of neat 2-methyl-3-butyn-2-ol (MBY) (catalyst: pentlandite Fe_3_Ni_6_S_8_; room temperature; v_flow_ = 12 mL min^−1^; data were measured as duplicates and are represented as mean ± σ).

The electrode architecture was evaluated under more demanding conditions, including higher current densities of 160 and 240 mA cm^−2^ for MBY without a PTFE layer (Figure 5). At these conditions, FE_MBE_ dropped to 19% due to substrate diffusion limitations and increased competitive hydrogen evolution. Potassium crossover averaged 18%, while water crossover increased to 15% and 27% at these higher densities due to greater cathode polarization. The more impermeable **M**_**dense**_ layer mitigated these effects, reducing potassium crossover to 1% and 3%, and water crossover to 7% and 12% for the respective current densities. Notably, in the configuration with **M**_**dense**_, FE_MBE_ remained stable at 18% when the current density was doubled from 80 to 160 mA cm^−2^.

Electrolysis was proven feasible at lower MBY concentrations of 0.1 M, 1 M, and 3 M in aqueous solutions at 80 mA cm^−2^ (Table S6). Interestingly, an optimum FE_MBE_ of 50% is visible at 3 M followed by 33% for 1 M and 17% for 0.1 M, e.g., comparing to 58% FE_MBE_ at 1 M on Cu-nanoarrays at 1300 mA cm^−2^ obtained by Bu *et al.* (Table S7).^49^ This again proves the adaptability of the presented SDE, while the observed optimum suggests that a tight adjustment of the local water to substrate ratio is imperative for optimal SDE function, which was also implied by the previously discussed results. Along the line of future developments, this optimum ratio of local substrate and water concentrations could be determined *via* *operando* spectroscopy and a broader range of materials as transport layers could be screened to implement this ratio experimentally, potentially supported by computational predictions. The same approach may be taken in order to simultaneously minimize water and electrolyte crossover.

The **M**_**dense**_ layer also allowed stable electrolysis of neat MBY for 22 h with a palladium catalyst, achieving an FE of 36%, which corresponded to an MBY conversion of 79% and an MBE yield of 60% (Figure S7). This result implies that the previously discussed changes in the SEM-EDX data for PTFE-bound catalyst layers in the electrolysis of MBY (Figures S3 and S6) are either not detrimental to long-term performance or that they are limited to pentlandite as a catalyst. The increased FE for undesired full hydrogenation to 2-methyl-2-butanol (MBA), recorded at 23%, is inherent to palladium and not a result of SDE functionality.^25^ The voltage increase of 64 mV h^−1^ and the water crossover rate of 58% do not exclude long-term applicability of the electrode but warrant extended optimization of the electrode’s stability.

### Conclusion

This study introduces the substrate diffusion electrode (SDE) as a concept for the electrochemical conversion of neat organic substrates. It eliminates the crossover of the organic substrate into the electrolyte, while the integration of a hydrophobic separation layer significantly reduces water crossover rates compared to configurations lacking this layer. The faradaic efficiency for semi-hydrogenation remains nearly constant for the most active palladium catalyst, with maximum Faraday efficiencies of 47% for MBY, 79% for MPY, and 21% for OCT. These results suggest that optimizing the SDE components in interplay with the used catalyst enhances hydrogenation outcomes for substrates with varying water solubility and viscosity. The electrode is capable to accomplish the desired transformation of MBY at 160 mA cm^−2^, with potassium and water crossover rates of 1% and 7%, respectively. The semi-hydrogenation efficiency of MBY remains stable over 22 h, reinforcing this potential. The SDE offers a promising foundation for further research into long-term stable electrochemical hydrogenation of neat organic substrates, potentially eliminating issues related to neat substrate feeds and electrode instability.

### Limitations of the study

The observed optima of the semi-hydrogenation FE for certain combinations of hydrophobic transport layer and substrate and the optima for specific substrate concentrations in the case of MBY and the unexpected FE trends in the screening of current densities hint at a tunable balance of local water and substrate concentrations. This balance and hence hydrogenation performance is assumed to be influenced by the choice of materials, the properties of the substrate feed and process conditions. However, this assumption alongside the concise reaction mechanism on a molecular level could not be directly verified in this study. Also, while substrate crossover to the adjacent electrolyte compartment could be eliminated, water and electrolyte crossover to the substrate compartment still occurs requiring further optimization of the system. In the context of future developments, the optimum concentration balances could be quantified by *operando* spectroscopy and potentially predicted computationally. The thus determined optimum local conditions could then be intertwined with a broader screening of materials and process conditions to implement these conditions experimentally. Molecular mechanisms could also be elucidated computationally and *operando*-spectroscopically along this line. With respect to the water and electrolyte crossover, a similar approach can be taken by conducting a broader screening of hydrophobic and hydrophilic transport layer materials and relating the results to *operando*-spectroscopic observations on local concentration balances and simulations of substrate and electrolyte transport. The combined results can be used for finding optimum material configurations minimizing crossover, while optimizing performance.

## Resource availability

### Lead contact

Requests for resources and procedures should be directed to the lead contact, Ulf-Peter Apfel (ulf.apfel@ruhr-uni-bochum.de).

### Materials availability

The experiments in this study did not generate new reagents.

### Data and code availability

• All data reported in this paper will be shared by the lead contact upon request.
• This paper does not report original code.
• Any additional information required to reanalyze the data reported in this paper is available from the lead contact upon request.

## Acknowledgments

J.W. and K.P. express their gratitude for the PhD fellowships provided by the *Studienstiftung des deutschen Volkes* and the *Fonds der Chemischen Industrie*, respectively. J.T.K. was funded by the 10.13039/501100001659Deutsche Forschungsgemeinschaft (DFG, German Research Foundation) under Germany’s Excellence Strategy – EXC2033-390677874-RESOLV. D.S. acknowledges financial support from the 10.13039/501100002347Bundesministerium für Bildung und Forschung (BMBF, Federal Ministry of Education and Research) through the NanoMatFutur Project “H2Organic” (no. 03XP0421) and the project BEFuel (no. 031B1403A).

## Author contributions

Conceptualization and methodology: J.W., K.P., J.T.K., L.W., D.S., U.-P.A.; execution of experiments: J.W., F.S., and Z.A.; data analysis and curation; J.W., F.S., and Z.A.; writing-original paper draft: J.W., K.P., J.T.K., L.W., D.S., and U.-P.A., funding acquisition and supervision: D.S. and U.-P.A. All authors have read and agreed to the published version of the manuscript.

## Declaration of interests

The authors declare no competing interests.

## Declaration of generative AI and AI-assisted technologies in the writing process

During the preparation of this work, the authors used ChatGPT (*OpenAI*) to proof-read the main text. After using this tool, the authors reviewed and edited the content as needed and take full responsibility for the content of the published article.

## STAR★Methods

### Key resources table

**Table:** 

REAGENT or RESOURCE	SOURCE	IDENTIFIER
**Chemicals, peptides, and recombinant proteins**		

Pentlandite catalyst Fe_3_Ni_6_S_8_	Custom mechanochemical synthesis^47^	N/A
Palladium-Pulver, APS 0.35–0.8 Micron, 99.95% (Metallbasis)	Fisher Scientific	Cat#15497495
Silver nanopowder, <150 nm particle size, 99% trace metals basis	Sigma Aldrich	Cat#484059
60 wt % PTFE Dispersion TFE-DISP30	QuinTech	Cat#13097
Nafion Dispersion D2020CS	Supplier: QuinTech Producer: Chemours	Chemours Product Identifier D12707882
Sustainion-XA9 Alkaline Ionomer 5% in ethanol	Dioxide Materials	SKU 68740
Sustainion XC-1 Alkaline Ionomer 5% in ethanol	Dioxide Materials	N/A
Triton X-100	Sigma-Aldrich	Cat#X100
Kynar Flex Ultraflex B Polyvinylidenedifluoride (PVDF)	Arkema	N/A

**Other**		

Electrode Support: H23	Freudenberg	SKU 31070009
Electrode Support: H23i2C6	Freudenberg	SKU 31070009
Electrode Support: H23i2	Freudenberg	SKU 31070009
PMV10 – POREX Virtek® PTFE Hydrophobic Venting Porous Membrane Sheets	Porex Technologies	N/A
PMV27 – POREX Virtek® PTFE Hydrophobic Venting Porous Membrane Sheets	Porex Technologies	N/A
Filtermembranen	Bola	N1690-65
Filtermembranen	Bola	N1617-55
Membranfilter, TE 38, 5μm	Cytiva	Cat#1190301
multiflon	Fluortex	N/A
multiflon TE-13	Fluortex	N/A
Nafion XL (27 μm)	Producer: Chemours Supplier: IonPower	N/A
Nafion HP (20 μm)	Producer: Chemours Supplier: IonPower	N/A
Nafion NR212 (51 μm)	Producer: Chemours Supplier: IonPower	N/A
Nafion 115 (127 μm)	Producer: Chemours Supplier: IonPower	N/A

### Method details

#### Electrode materials and fabrication

If not stated otherwise, all materials were supplied by commercial vendors without further purification. 2-Methyl-3-butyn-2-ol (MBY) and 3-methyl-1-pentyn-3-ol (MPY) as hydrogenation substrates were purchased from *Sigma Aldrich****,*** 3,7-dimethyl-6-octen-1-yn-3-ol (OCT) from *TCI Chemical*. *Sigma Aldrich* supplied the silver nanoparticle catalyst while the palladium microparticle catalyst was purchased from *Fisher Scientific*. Sustainion-XA9 and Sustainion XC-1 as polymer binders were supplied by *Dioxide Materials*. Nafion D2020CS polymer binder dispersion in alcoholic solvent was purchased from *Ionpower* and processed as described in literature.^25^ TFE-DISP30 PTFE-dispersion as polymer binder was purchased from *Quintech* and processed as described in literature.^25^ Polyvinylidenedifluoride (PVDF) polymer binder was purchased from *Arkema*. Nafion membranes as hydrophilic transport layers were manufactured by *Chemours*. **M**_**wide**_ was supplied by *Bola* and **M**_**dense**_ by *Porex Technologies*, as hydrophobic transport layers. FM-FAA-3-PK 130 as separating membrane in the zero-gap hydrogenation of MPY and MBY (Table S6) was supplied by *Fumatech* and used after conditioning in 1 M KOH in H_2_O for 24 h.

The pentlandite catalyst Fe_3_Ni_6_S_8_ was synthesized mechanochemically according to established procedures.^25^ Palladium microparticles (0.35-0.80 μm) and silver nanoparticles (<150 nm), used due to their feasibility proven in past studies,^27^ were commercially sourced and used without further treatment. Pentlandite and palladium electrodes for MBY experiments contained PTFE as a binder, while those for MPY and OCT used Nafion. Silver electrodes used PVDF as a binder for all substrates, since its feasibility was shown in past studies.^27^ PTFE- and PVDF-containing electrodes were manufactured using established methods.^25^^,^^27^^,^^36^

To prepare the catalyst inks containing PTFE, 0.5 g of the respective catalyst was combined with 15 g of 2-propanol, 4 mL of H_2_O, and 0.2 g of Triton X-100. The resulting mixture was sonicated in an ultrasonic bath for 5 minutes. Following this, the ink was dispersed using a T 25 digital Ultra-Turrax by *IKA* at 13,600 rpm for 1 minute. Next, the required amount of a 60 wt% PTFE dispersion was added while stirring. The suspension was then uniformly spray-coated using an *Iwata* SBS airbrush onto an 8.5 x 8.5 cm carbon paper, which had been preheated to 95°C on a hot plate. The PTFE-containing electrodes were subsequently heat-treated at 240°C for 20 minutes to remove the surfactant in the ink. For the PVDF-containing ink, a 5 wt% PVDF solution in acetone was incorporated into a mixture containing 0.5 g of silver nanoparticles in 15 g of methanol to achieve the desired binder concentration. The ink was then evenly spray-coated onto an 8.5 x 8.5 cm carbon paper, which had been heated to 65°C on a hot plate. No further processing steps were required for the PVDF-containing inks. Compared to the PTFE protocol, the Nafion-bound electrodes feature few differences in preparation: i) The binder is added before instead of after ultrasonication, ii) no surfactant is incorporated into the catalyst ink and iii) the electrodes are not heat-treated before use.

A catalyst loading of 2 mg cm^-2^ was employed throughout. H23 carbon paper (*Freudenberg*) supported electrodes were used for MPY and OCT, and H23i2 for MBY. Spray-coated electrodes were cut into 40 mm diameter circles and hot-pressed onto 58 mm diameter Nafion 212 membranes at 135°C and 10 bar. These compound electrodes were soaked in ultrapure water for 5 min before use.

#### Cell setup and measurement protocol

Electrochemical investigations were conducted in a custom-built electrolyzer adapted from previous studies.^25^ The cell comprises stainless steel end plates including connections for the tubing through which the cell was supplied with substrate on one side and anolyte on the other side. PTFE insulators insulated the cell from the adjacent copper current collector plates. The latter were connected to the potentiostat and supplied constant current to the adjacent titanium serpentine flow fields on the substrate and anode side. The substrate and catholyte compartments were separated by the SDE compound electrode, while the catholyte and anolyte compartments were separated by a Nafion 115 membrane. The catholyte central compartment consisted of a serpentine titanium flow field open on both sides that faced the substrate diffusion electrode on the substrate-facing side and the Nafion 115 membrane on the anode-facing side. The catholyte central compartment had two opposite nozzles for the connection of the tubing oriented towards the sides of the electrolyzer. 2 M KOH in H₂O was used as both catholyte and anolyte, with compressed nickel foam as the anode. The geometric surface area of the SDE exposed to the substrate and electrolyte with the catalyst layer was 7.1 cm^-2^. Viton and PFA tubing was used to recirculate the neat organic substrate and the electrolytes through the cell with a rotating-piston peristaltic pump.

### Quantification and statistical analysis

Dynamic vapor sorption (DVS) measurements were performed on a DVS Resolution (Dual Vapor Gravimetric Sorption Analyzer) by *Surface Measurement Systems* equipped with a *Cahn* ultra-micro scale coupled with a video camera. Contact angles were determined by placing a drop of 50 μl water on the respective surface, waiting for 1 min and recording images from a 90° angle using a VHX-6000 digital microscope by *Keyence*. Scanning electrode microscopy (SEM) imaging was performed on a *ZEISS* Gemini2 Merlin HR-FESEM equipped with an *OXFORD* AZtecEnergy X-ray microanalysis system for energy dispersive X-ray spectroscopy (EDX). Potassium was quantified *via* a *Spectro Arcos* optical emission spectrometer with inductively coupled plasma. The water content of the product mixtures was determined by Karl-Fischer titration with a KFT-Titrino 795 by *Metrohm*. The hydrogenation products of MPY- and OCT-containing samples were analyzed with a GCMS-FID 6890N/5975B by *Agilent* equipped with an *Agilent* DB-Wax 60 m∗0.25 mm∗0.5 μm column, those containing MBY with a *Shimadzu* GC-MS QP2020 equipped with a Supelco Carboxen 1010 Plot column and a HS20 headspace sampler. Contents of the hydrogenation products were determined by integration of the respective chromatogram peaks with MassHunter software by *Agilent* for MPY and OCT and with Postrun Analysis by *Shimadzu* for MBY. Calibration was performed using external standard solutions consisting of 200 ppm, 400 ppm, 600 ppm, 800 ppm and 1000 ppm alkene and alkane with relation to the total volume of the sample, respectively, in H_2_O for MBY and in ethanol for MPY and OCT. The voltage increase of the 22 h electrolysis run was determined from the difference between the mean voltage during the first 30 min and the last 30 min of the experiment. The provided data was measured in duplicates and is presented as the mean ± σ (population standard deviation), as given in the figure legends. OriginPro 2022 by *OriginLab Corporation* was used for statistical analysis of the primary data. The data for FE, potassium and water crossover was rounded to zero decimal places.
