# Supplementary material for: Substrate diffusion electrodes allow for the electrochemical hydrogenation of concentrated alkynol substrate feeds
Source: iScience. 2025 Jan 10;28(2):111789. doi: 10.1016/j.isci.2025.111789 (PMC11803216; doi:10.1016/j.isci.2025.111789)
Supplement: Document S1. Figures S1–S10 and Tables S1–S7 [file mmc1.pdf]

iScience, Volume *28*

Supplemental information

Substrate diffusion electrodes allow for the electrochemical hydrogenation of concentrated alkynol substrate feeds

Jonas Wolf, Fatima Shahrour, Zafer Acar, Kevinjeorjios Pellumbi, Julian Tobias Kleinhaus, Leon Wickert, Ulf-Peter Apfel, and Daniel Siegmund

Table S1. Screening of PTFE-membranes as hydrophobic layers within the Substrate Diffusion Electrode (SDE)

Faradaic efficiency (FE) for the hydrogenation of 2-methyl-3-butyn-2-ol (MBY) to 2-methyl-3-buten-2-ol (MBE) and 2-methylbutan-2-ol (MBA) with varying pore size and thickness of the respective PTFE membrane ($Fe_3Ni_6S_8$ catalyst, conditions: s. manuscript, data were measured as duplicates and are represented as mean; related to **Figure 3**).

Trade name (supplier)	Pore size (µm)	Thickness (mm)	FE_{MBE} (%)	FE_{MBA} (%)	Comment
-	-	-	38	6	no PTFE-membrane used
-[a]	-	-	41	0	no PTFE-membrane used
Virtek PMV10 (*Porex Technologies*)	0.5[b]	0.13	13	2	-
N1690-65 (*Bola*)	10	1	13	1	-
N1617-55 (*Bola*)	25	1	8	1	-
Virtek PMV27 (*Porex Technologies*)	0.1[b]	0.19	4	1	-
TE 38 (*Cytiva*)[a]	5	1	1	0	little observable product due to insufficient substrate permeability
multiflon (*Fluortex)*	0.2	0.07	-	-	quantitative crossover of the compartments into each other
multiflon TE-13 (*Fluortex)*	0.2	0.035	-	-	quantitative crossover of the compartments into each other

[a]silver as catalyst

[b]given by the supplier as the filtration efficiency > 99.9%

Table S2. Screening of Nafion-membranes as hydrophilic layers within the Substrate Diffusion Electrode (SDE)

Faradaic efficiency (FE) for the hydrogenation of 2-methyl-3-butyn-2-ol (MBY) to 2-methyl-3-buten-2-ol (MBE) and 2-methylbutan-2-ol (MBA) ($Fe_3Ni_6S_8$ catalyst, conditions: s. manuscript, data were measured as duplicates and are represented as mean; related to **Figure 3**).

**Thickness (µm)**	**FE_{MBE} (%)**	**FE_{MBA} (%)**	**Water crossover (%)[a]**	**Potassium crossover (%)[b]**
20	39	10	17	17
27	30	7	10	27
51	38	10	7	18
127	5	1	0	-

[a]given as the percentage of water in the product mixture after 1 h electrolysis

[b]given as the percentage of the consumed charge that is equilibrated by the transport of a potassium ion through the Nafion-membrane

Table S3. Screening of carbon supports and polymer binders within the Substrate Diffusion Electrode (SDE)

Faradaic efficiency (FE) for the semi-hydrogenation of 2-methyl-3-butyn-2-ol (MBY), 3-methyl-1-pentyn-3-ol (MPY) and dehydrolinalool (OCT) ($Fe_3Ni_6S_8$ catalyst, conditions: s. manuscript, data were measured as duplicates and are represented as mean; related to **Figure 3**).

Carbon support	Binder	Substrate	FE_{alkene} (%)	Water crossover (%)[a]	Comment
H23	PTFE	MBY	34	15	
H23i2C6	PTFE	MBY	34	8	
H23i2	PTFE	MBY	38	7	
H23i2	PVDF	MBY	26	-	
H23i2	Sustainion XA-9	MBY	18	-	
H23i2	Nafion	MBY	-	-	decomposition of catalyst layer
H23	XC-1	MPY	-	-	decomposition of catalyst layer
H23	Nafion	MPY	13	9	-
H23	PVDF	MPY	12	9	
H23	PTFE	MPY	-	-	decomposition of catalyst layer
H23i2	Nafion	MPY	2	9	-
H23	Nafion	OCT	4	9	-
H23i2	Nafion	OCT	1	9	-
H23	PTFE	OCT	-	-	decomposition of catalyst layer

[a]given as the percentage of water in the product mixture after 1 h electrolysis

[b]given as the percentage of the consumed charge that is equilibrated by the transport of a potassium ion through the Nafion-membrane

Table S4. **Screening of combinations of catalyst, hydrophobic transport layer and substrate**

Cell voltages U_{cell}, Faraday efficiencies (FE) for alkane formation, 2-methyl-3-buten-2-ol yield (MBE yield) and reaction rate (MPY = 3-methyl-1-pentyn-3-ol; OCT = dehydrolinalool; data were measured as duplicates and are represented as mean; related to **Figure 4**).

Entry	Cat.	Substrate	PTFE	MBE yield (%)	Reaction rate	FE alkane	U_{cell} (V)
1	Pd	MBY	M_{wide}	<1	5.0	1	4.29
2	Pd	MPY	M_{wide}	5	4.3	7	4.28
3	Pd	OCT	M_{wide}	<1	4.0	1	3.76
4	Pd	MBY	M_{dense}	4	4.4	4	4.72
5	Pd	MPY	M_{dense}	2	1.8	3	3.73
6	Pd	OCT	M_{dense}	<1	1.5	3	4.02
7	Ag	MBY	M_{wide}	1	0.6	0	4.21
8	Ag	MPY	M_{wide}	2	0.8	0	5.46
9	Ag	OCT	M_{wide}	<1	1.4	0	5.65
10	Ag	MBY	M_{dense}	1	8.4	0	4.18
11	Ag	MPY	M_{dense}	1	4.2	0	4.21
12	Ag	OCT	M_{dense}	<1	1.3	0	3.64
13	Pn	MBY	M_{wide}	1	2.8	1	4.52
14	Pn	MPY	M_{wide}	<1	1.3	0	4.24
15	Pn	OCT	M_{wide}	<1	0.2	0	4.83
16	Pn	MBY	M_{dense}	1	6.9	2	2.80
17	Pn	MPY	M_{dense}	<1	2.5	0	3.96
18	Pn	OCT	M_{dense}	<1	0.2	0	3.05
19	Pd	MBY	None	4	2.2	3	4.51
20	Pd	MPY	None	7	0.6	4	3.93
21	Pd	OCT	None	2	0.4	3	5.61
22	Ag	MBY	None	3	2.3	0	4.72
23	Ag	MPY	None	3	0.3	0	4.3
24	Ag	OCT	None	<1	0.1	0	5.24
25	Pn	MBY	None	3	0.6	6	3.2
26	Pn	MPY	None	1	0.3	0	4.90
27	Pn	OCT	None	<1	0.1	0	4.46

Table S5. Comparison of electrode concepts for the electrocatalytic semi-hydrogenation of 2-methyl-3-butyn-2-ol (MBY)

Faraday efficiency and catalyst mass activity of literature-known protocols and this work (data were measured as duplicates and are represented as mean, related to **Figure 4**).

Electrode	Substrate Concentration (M)	Current density (mA cm^{-2})	FE_{MBE} (%)	Catalyst mass activity (g g_{cat}^{-1} h^{-1})
Palladium-coated Substrate Diffusion Electrode (this work)	Neat	80	47	30.0
Pentlandite-coated Substrate Diffusion Electrode (this work)	Neat	80	38	24.3
Pentlandite-coated Substrate Diffusion Electrode (this work)	3	80	50	31.9
Ag-plated CF (Apfel *et al.*)[27]	1	240	76	1465.4
Pentlandite-coated CF (Apfel *et al.*)[25]	1	240	70	54.0
Cu-nanoarrays (Zhang *et al.*)[47]	1	1300	58	357.0

Table S6. **Comparative semi-hydrogenation of 3-methyl-1-pentyn-3-ol (MPY) and dehydrolinalool (OCT) in a zero-gap electrolyzer**

Faradaic efficiencies of the alkene formation upon usage of a FM-FAA-3-PK-130 anion-exchanging separator membrane, a nickel foam anode and Nafion-bound carbon paper cathodes with varying catalysts in aqueous solution with differing contents of ethanol (active area: 12.57 cm^2; current density: 80 mA cm^{-2}; run time: 1 h; electrode preparation according to the protocol used for the Substrate Diffusion Electrode (SDE); data were measured as duplicates and are represented as mean; related to **Figure 4**).

Substrate	Catalyst	Ethanol content (vol%)	FE_{alkene} (%)
MPY	Pd	10	50
MPY	$Fe_3Ni_6S_8$	10	35
OCT	Pd	50	9
OCT	$Fe_3Ni_6S_8$	50	0

Table S7. Screening of 2-methyl-3-butyn-2-ol (MBY) concentrations

Cell voltages U_{cell} and Faraday efficiencies (FE) for the formation of 2-methyl-3-buten-2-ol (MBE) and 2-methylbutan-2-ol (MBA) on a Substrate Diffusion Electrode (SDE) (catalyst: $Fe_3Ni_6S_8$; binder: 10 wt% PTFE; carbon support: H23i2; data were measured as duplicates and are represented as mean; related to **Figure 4**).

MBY concentration (M)	FE_{MBE} (%)	FE_{MBA} (%)	U_{cell}
0.1	17	1	3.74
1	33	5	5.27
3	50	8	3.27
neat	38	6	3.20

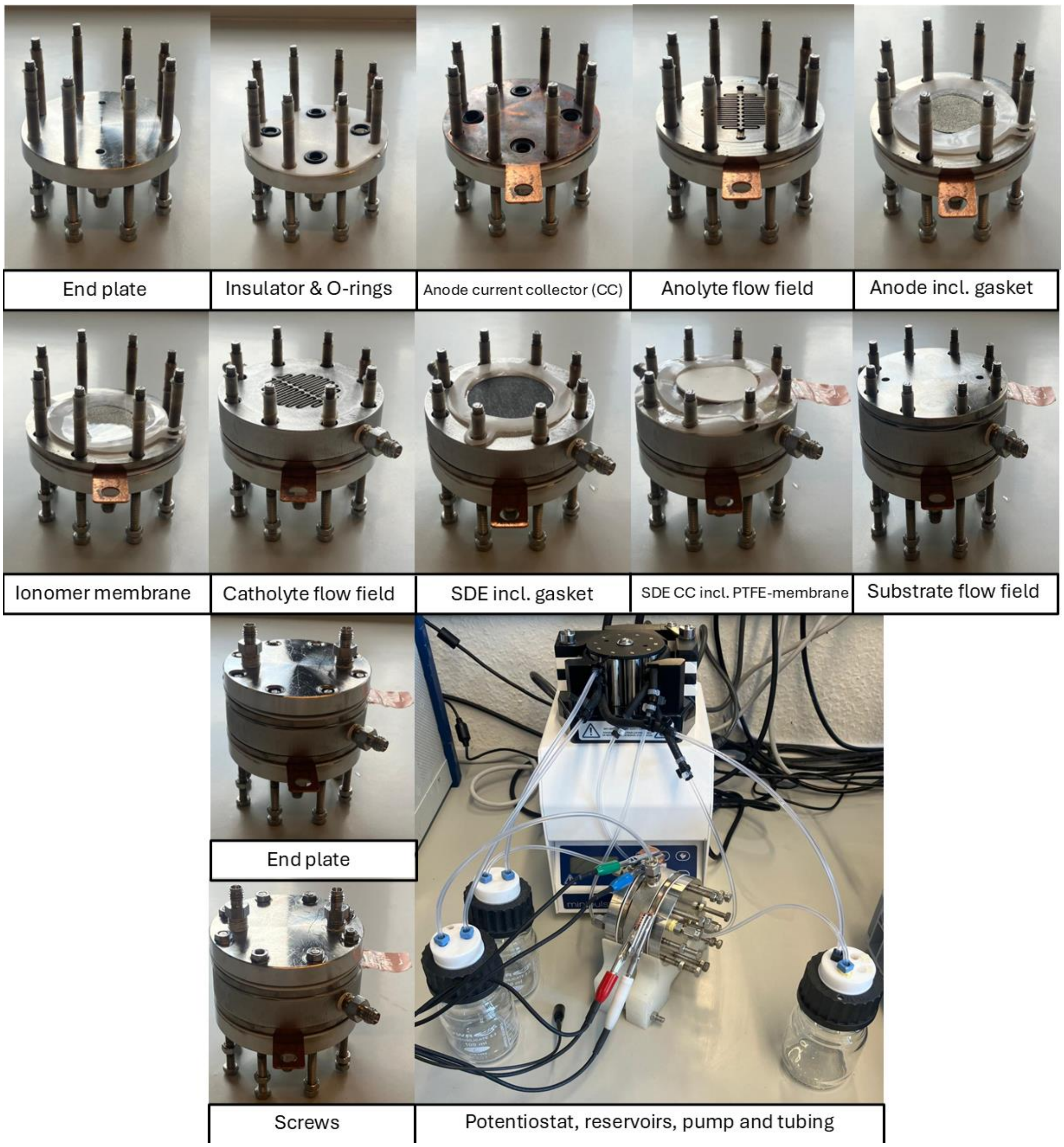

Figure S1. Assembly of the electrochemical flow reactor setup incorporating the Substrate Diffusion Electrode (SDE)

For the setups incorporating a PTFE-membrane as hydrophobic separation layer, a current collector consisting of copper tape glued onto a PTFE gasket was used and brought into direct contact with the carbon porous transport layer (related to **Figure 1**).

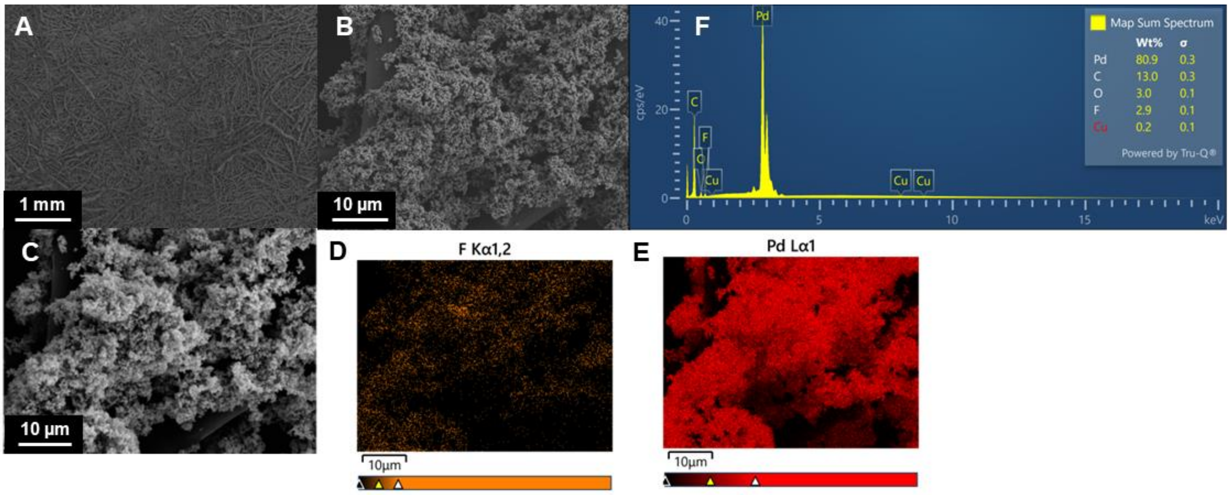

Figure S2. SEM- and EDX data of palladium-coated H23 carbon paper with 10 wt% Nafion as polymer binder

Images on the millimeter and micrometer scale (A, B, C), EDX maps (D, E) and EDX spectrum (F) (related to **Figure 4**).

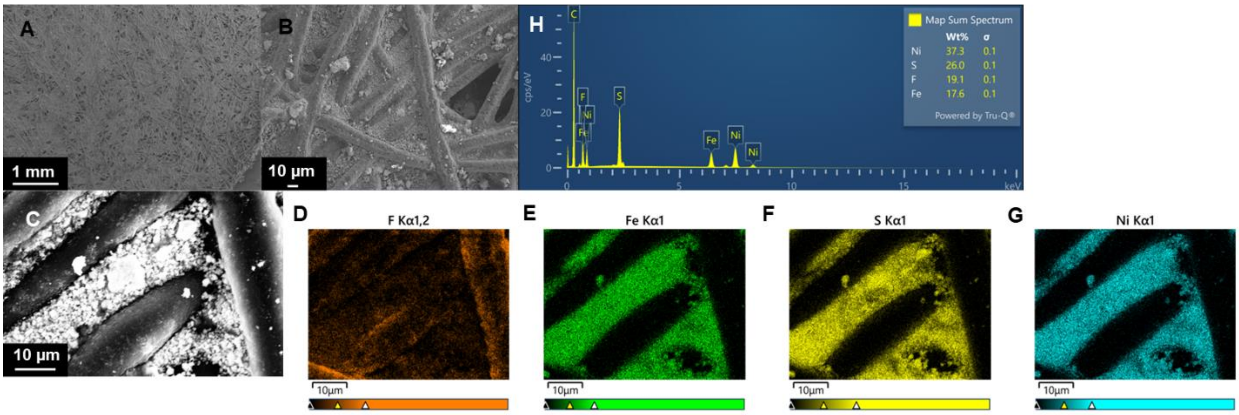

Figure S3. SEM- and EDX data of $Fe_3Ni_6S_8$-coated H23i2 carbon paper with 10 wt% PTFE as polymer binder

Images on the millimeter and micrometer scale (A, B, C), EDX maps (D, E, F, G) and EDX spectrum (H) (related to **Figure 4**).

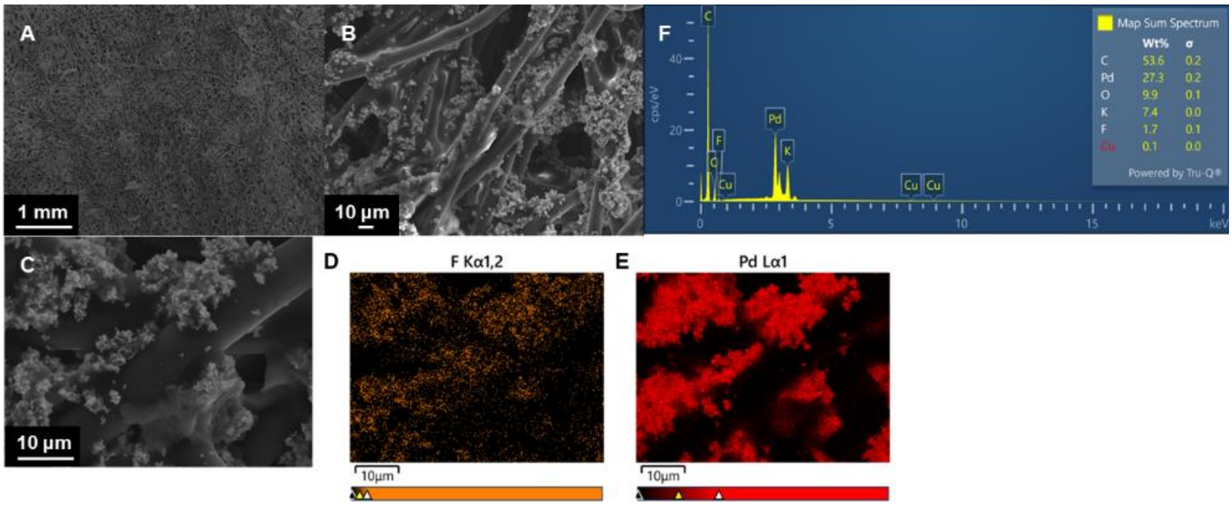

Figure S4. SEM- and EDX data of palladium-coated H23 carbon paper with 10 wt% Nafion as polymer binder after electrolysis of neat 3-methyl-1-pentyn-3-ol (MPY)

Images on the millimeter and micrometer scale (A, B, C), EDX maps (D, E) and EDX spectrum (F). Electrolysis was performed for 1 h at 80 mA cm^{-2} using a Substrate Diffusion Electrode (SDE) (related to **Figure 4**).

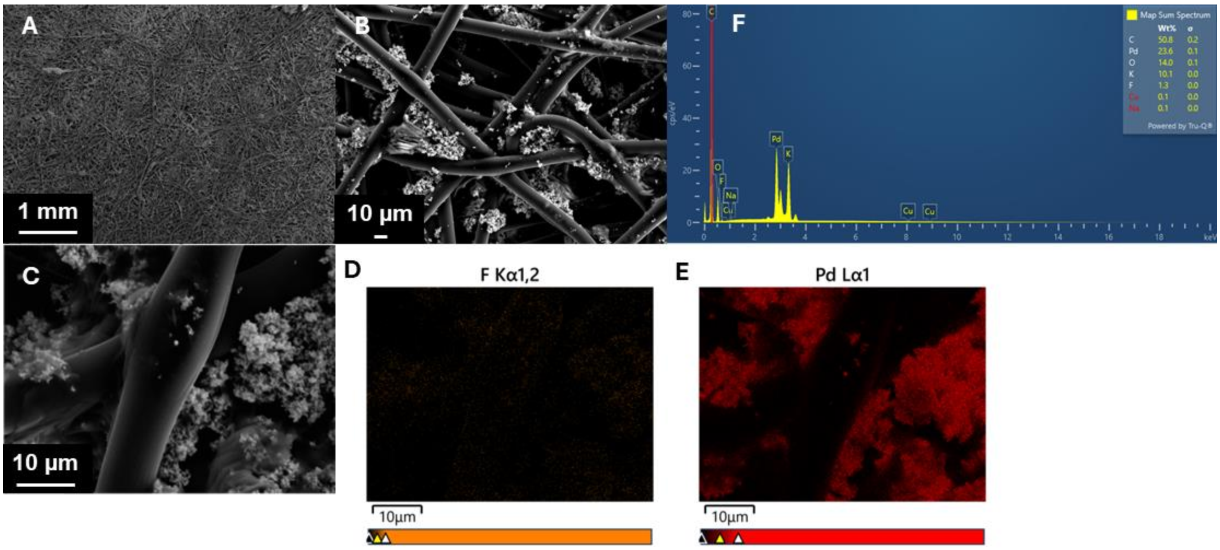

Figure S5. SEM- and EDX data of palladium-coated H23 carbon paper with 10 wt% Nafion as polymer binder after electrolysis of neat dehydrolinalool (OCT)

Images on the millimeter and micrometer scale (A, B, C), EDX maps (D, E) and EDX spectrum (F). Electrolysis was performed for 1 h at 80 mA cm^{-2} using a Substrate Diffusion Electrode (SDE) (related to **Figure 4**).

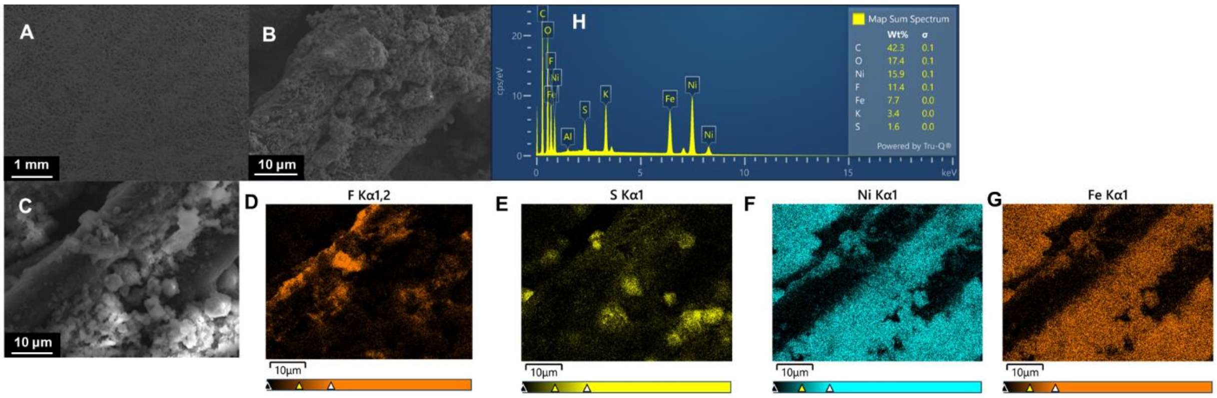

Figure S6. SEM- and EDX data of $Fe_3Ni_6S_8$-coated H23i2 carbon paper with 10 wt% PTFE as polymer binder after the electrolysis of neat 2-methyl-3-butyn-2-ol (MBY)

Images on the millimeter and micrometer scale (A, B, C), EDX maps (D, E) and EDX spectrum (F). Electrolysis was performed for 1 h at 80 mA cm^{-2} using a Substrate Diffusion Electrode (SDE) (related to **Figure 4**).

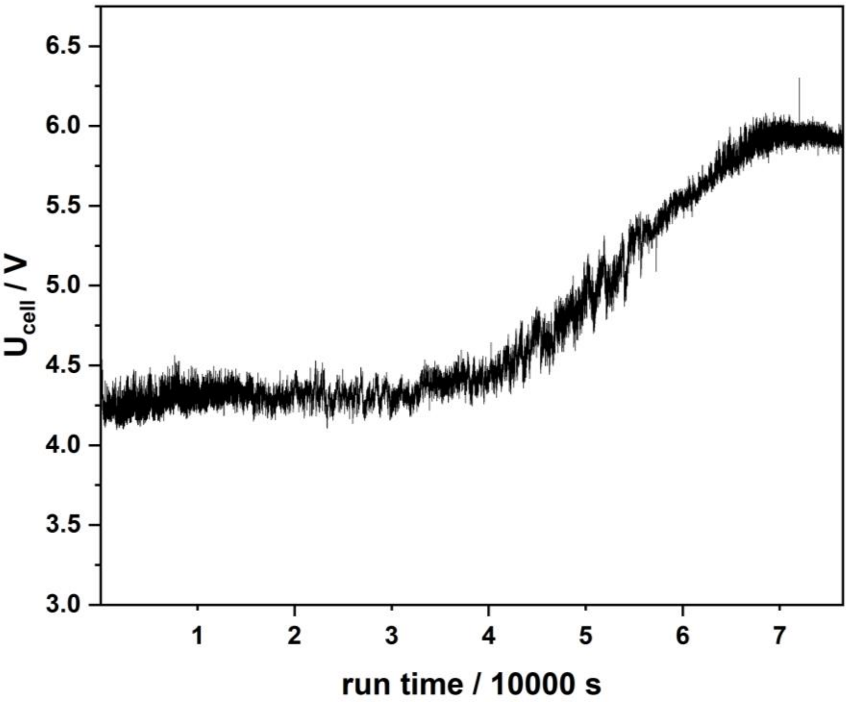

Figure S7. Voltage curve of the electrolysis of neat 2-methyl-3-butyn-2-ol (MBY) for 22 h

Electrolysis was performed on a palladium-coated Substrate Diffusion Electrode (SDE) (polymer binder: 10 wt% PTFE; carbon support: H23i2; current density: 80 mA cm^{-2}; related to **Figure 5**).

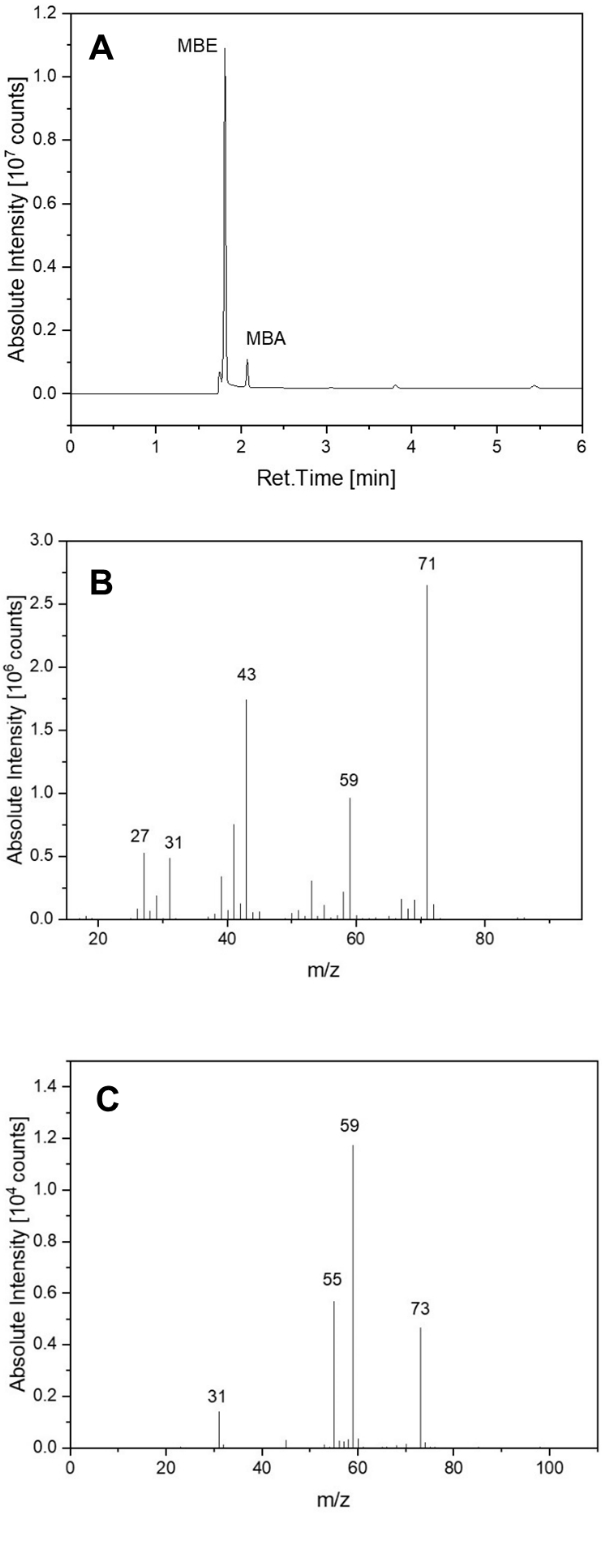

Figure S8. Analysis data of an exemplary crude product sample from the semi-hydrogenation of neat 2-methyl-3-butyn-2-ol (MBY)

Gas chromatogram (A) and the corresponding mass spectra belonging to the hydrogenation products 2-methyl-3-buten-2-ol (MBE) (B) and 2-methylbutan-2-ol (MBA) (C) (comparable to previous studies[27]; (related to **Figure 3, 4, 5**).

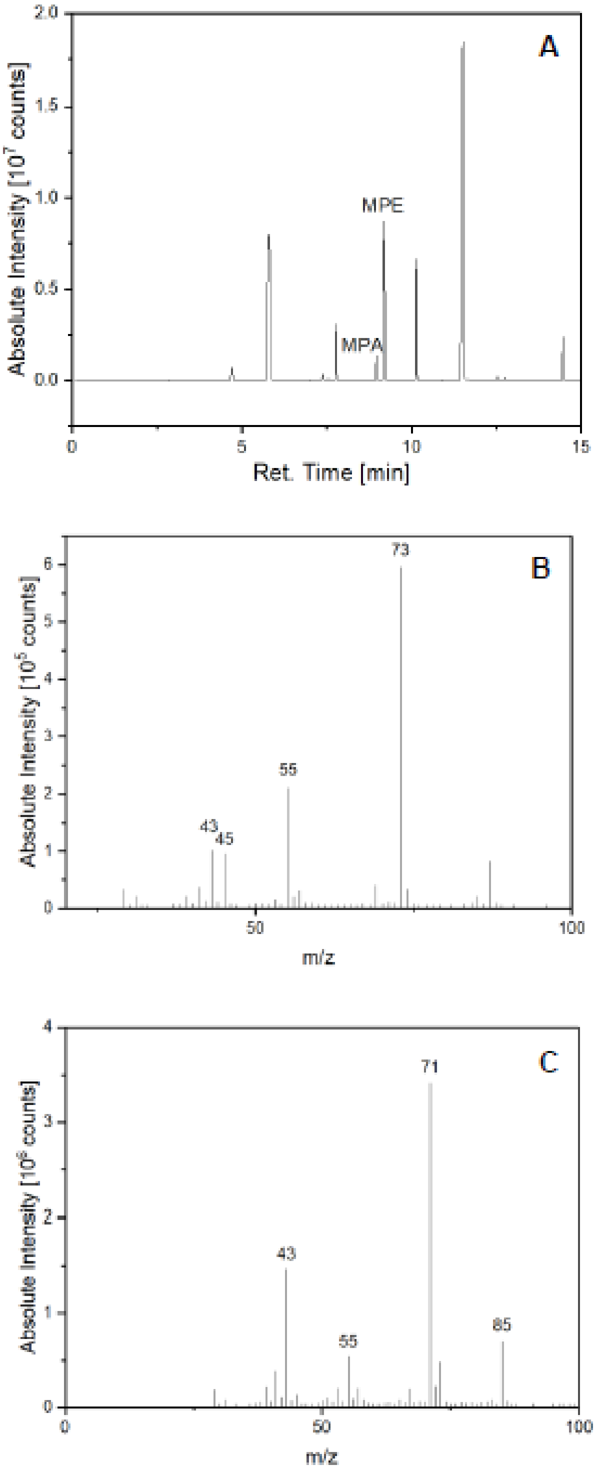

Figure S9. Analysis data of an exemplary crude product sample from the semi-hydrogenation of neat 3-methyl-1-pentyn-3-ol (MPY)

Gas chromatogram (A) and the corresponding mass spectra belonging to the hydrogenation products 3-methyl-1-penten-3-ol (MPE) (B) and 3-methyl-3-pentanol (MPA) (C) (remaining peaks were shown to be artifacts of the measurement and to not correspond to actual organic compounds; related to **Figure 4**).

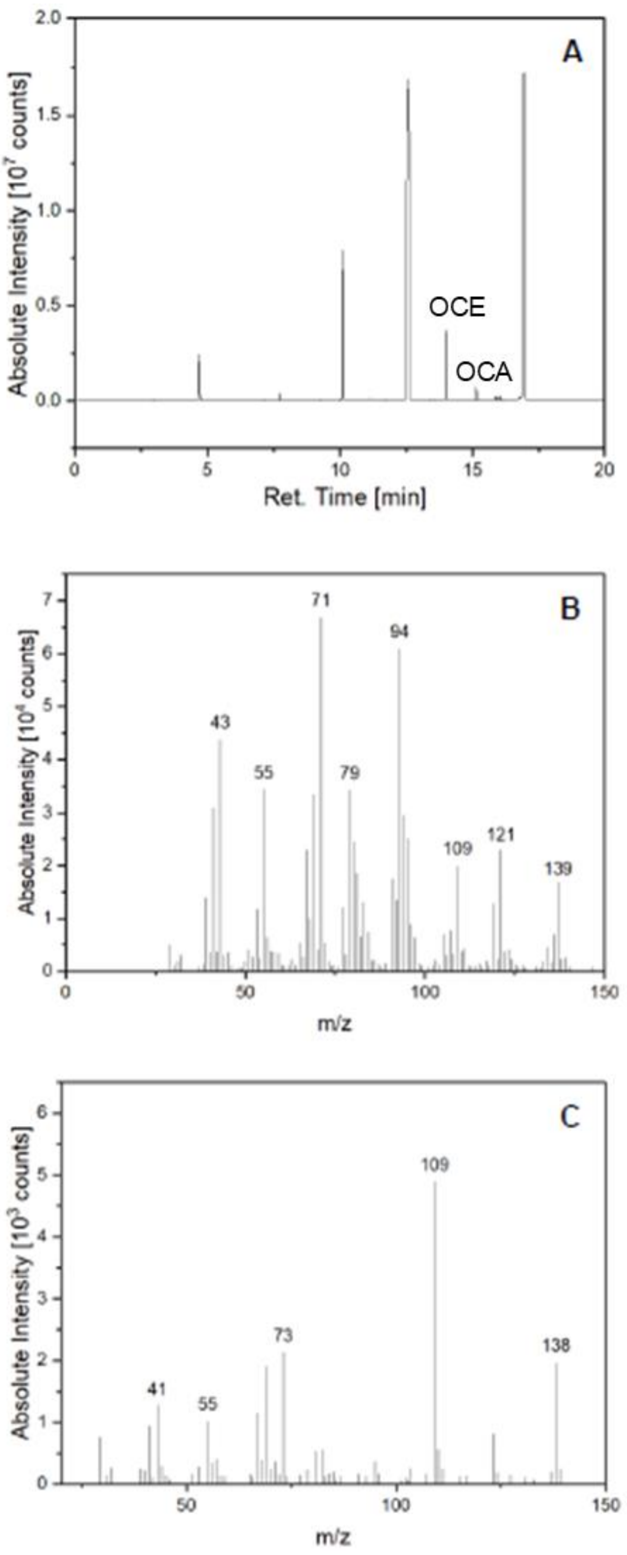

Figure S10. Analysis data of an exemplary crude product sample from the semi-hydrogenation of neat dehydrolinalool (OCT)

Gas chromatogram (A) and the corresponding mass spectra belonging to the hydrogenation products linalool (OCE) (B) and dihydrolinalool (OCA) (C) (comparable to previous studies[27]; remaining peaks were shown to be artifacts of the measurement and to not correspond to actual organic compounds; related to **Figure 4**).
